# Surgical Outcomes Stratified by Type of Transportation and Presence of Coronary Reperfusion in Patients with Coronary Malperfusion Caused by Type A Aortic Dissection

**DOI:** 10.5761/atcs.oa.24-00182

**Published:** 2025-02-01

**Authors:** Kazuki Noda, Yosuke Inoue, Yoshimasa Seike, Hitoshi Matsuda

**Affiliations:** Department of Cardiovascular Surgery, National Cerebral and Cardiovascular Center, Suita, Osaka, Japan

**Keywords:** coronary malperfusion, acute type A aortic dissection, coronary angiography, percutaneous coronary intervention, peripheral extracorporeal membrane oxygenation

## Abstract

**Purpose:** Owing to the time-sensitive nature of myocardial ischemia, challenging clinical scenarios should be considered in patients with type A acute aortic dissection (AAAD) complicated by coronary malperfusion. In clinical settings, the diagnosis and reperfusion strategies for coronary malperfusion often depend on institutional resources. This study evaluated early surgical outcomes in such patients, focusing on transportation type and clinical management.

**Methods:** We retrospectively reviewed 70 patients who underwent emergency surgery for AAAD with coronary malperfusion, excluding those with cardiac tamponade on arrival, between 1997 and February 2024. Patients were divided into 2 groups based on transportation: direct transfer and referral.

**Results:** Overall, in-hospital mortality was 27%, with only 1 of 9 patients surviving with preoperative peripheral extracorporeal membrane oxygenation (ECMO). Mortality and morbidity did not significantly differ between groups. Univariate analysis identified left coronary artery involvement and preoperative hemodynamic instability as significant risk factors. Additionally, preoperative diagnostic-only coronary angiography (CAG) with unsuccessful reperfusion was a potential risk factor (P = 0.06).

**Conclusions:** Regardless of transportation type, preoperative peripheral ECMO itself could not be a definitive solution in AAAD patients with coronary malperfusion. Also, patients who underwent preoperative CAG with unsuccessful reperfusion might be fatal, especially with suspected left coronary artery involvement.

## Introduction

Type A acute aortic dissection (AAAD) can result in a life-threatening clinical condition known as coronary artery malperfusion. Although its most effective treatment strategy is still not established, an early reperfusion strategy achieved by catheter revascularization before central aortic repair may improve the clinical outcomes of patients with coronary artery malperfusion due to AAAD.^1)^

The distinct feature related to coronary malperfusion is the time-sensitive nature of acute myocardial infarction (AMI), requiring early reperfusion to save the patients’ lives.^2)^ Japan has a well-established emergency medical services system; however, in actual clinical settings, coronary malperfusion caused by AAAD is sometimes difficult to diagnose, and patients are sometimes transferred to community hospitals with cardiology services but without cardiovascular surgical services as AMI. In addition, whether cardiologists need to perform early coronary reperfusion procedures in patients with coronary malperfusion caused by AAAD or to refer the patients to cardiac surgeons is unclear. Moreover, the actual effect of mechanical support, including peripheral extracorporeal membrane oxygenation (ECMO), on patient survival remains unclear in patients with AAAD.

This variability in transportation and management motivated us to investigate how different pathways affect patient outcomes. To our knowledge, no study has comprehensively examined the flow and types of transportation of patients to the hospital under these diverse clinical conditions.

With these considerations, this study aimed to investigate the early surgical outcomes of patients with coronary malperfusion caused by AAAD, focusing on the diagnosis and subsequent management of these patients, the appropriate timing and types of transportation from AAAD onset, and patient’s hemodynamics.

## Materials and Methods

### Patients and data collection

**Fig. 1:**
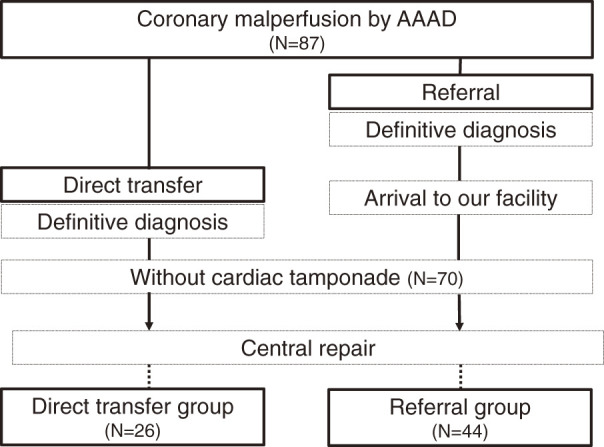
Flow chart of the enrollment process of the study participants. Following the exclusion of patients who did not meet the criteria for coronary malperfusion and who had cardiac tamponade on arrival, only 70 patients were included in the final analysis. AAAD: type A acute aortic dissection

Overall, 987 patients underwent emergency surgery for AAAD in our facility between January 1997 and February 2024. Data from 87 patients who also had coronary malperfusion were retrospectively reviewed. In all, 17 patients with cardiac tamponade on arrival were excluded. Finally, 70 patients with coronary malperfusion caused by AAAD without cardiac tamponade were enrolled. The patients were divided into the following 2 groups according to the transportation mode: direct transfer (N = 26, 37%) and referral (N = 44, 73%) groups (**Fig. 1**).

### Definitions and treatment policies

Based on the definitive diagnosis of AAAD, coronary malperfusion was defined as new ST-segment elevation at the J point in 2 contiguous leads with the cutoff point as greater than 0.1 mV on electrocardiography^3)^ and/or abnormality of wall motion on echocardiography.

In our facility, if AAAD had already been diagnosed on arrival, emergency coronary angiography (CAG) would not be performed despite strong suspicion of coronary malperfusion. If cardiac tamponade was present on arrival, emergency pericardiotomy, followed by a central aortic repair, was always preferred, even though a definitive diagnosis had not yet been made. Conversely, if CAG had already been performed for suspected AMI without the diagnosis or suspicion of AAAD, percutaneous coronary intervention (PCI) would be prioritized when coronary malperfusion was caused by AAAD. Throughout the study, our treatment strategy for managing patients with coronary malperfusion due to AAAD has remained consistent.

### Surgical techniques

A median sternotomy was employed for all surgical procedures for AAAD. The right axillary and common femoral arteries were the most frequently selected sites for arterial cannulation.^4,5)^ Cold blood cardioplegia was routinely administered retrogradely through the coronary sinus. If an intimal tear was identified inside the sinus of Valsalva, a composite graft root or valve-sparing aortic root replacement was performed using the reimplantation technique. Conversely, if the intimal tear was not located in the sinus of Valsalva, such as Neri’s classification type A,^6)^ proximal aortic stump reinforcement using surgical glue was indicated. Coronary artery bypass grafting (CABG) was added during cooling in patients with intraoperative myocardial ischemia not resolved by cardiopulmonary bypass (CPB). In cases with difficulty in CPB weaning or persistent wall motion abnormalities and/or indistinct blood flow around the coronary ostium as detected by intraoperative transesophageal echocardiography, additional CABG was occasionally required.

### Statistical analyses

EZR, a graphical user interface for R, was used for all statistical analyses (Saitama Medical Center, Jichi Medical University, Saitama, Japan; R Foundation for Statistical Computing, Vienna, Austria). Continuous variables were analyzed using the Mann–Whitney U-test for nonparametric analysis and presented as medians with interquartile ranges (IQR), whereas nominal variables were examined using Fisher’s exact test. Univariate analysis was conducted using the logistic regression analysis for estimation. For each analysis, a P-value of <0.05 was considered significant. All variables examined in the logistic regression analysis were included in **Supplementary File 1**. The cutoff points for the continuous variables were calculated using the shortest distance from the receiver-operating characteristic curve to the perfect corner.

## Results

**Table 1** shows a comparison of the patient’s demographics between the 2 groups.

**Table 1:** Pre and intra-operative patients’ demographics

Variables	Overall (N = 70)	Direct transfer (N = 26)	Referral (N = 44)	P-value
Age (years), means ± SD	65 ± 14	66 ± 11	64 ± 15	0.60
Male gender, n (%)	38 (54)	10 (38)	28 (64)	0.05
Past history				
Hypertension	62 (89)	22 (85)	40 (91)	0.41
Hyperlipidemia	24 (34)	8 (31)	16 (36)	0.61
Ischemic heart disease	12 (17)	5 (19)	7 (16)	0.75
Old cerebral vascular accident	4 (6)	2 (8)	2 (5)	0.63
Preoperative status, n (%)				
Shock	35 (50)	9 (35)	26 (59)	0.049
Coma	18 (26)	3 (12)	15 (34)	0.047
Under peripheral ECMO	9 (13)	3 (12)	6 (14)	1
CPR	19 (27)	6 (23)	13 (30)	0.59
Neck vessels dissection	11 (16)	5 (19)	6 (14)	0.735
Visceral malperfusion	4 (6)	1 (4)	3 (7)	1
Leg malperfusion	6 (9)	3 (12)	3 (7)	0.664
The culprit of coronary malperfusion				
Left coronary artery	35 (50)	17 (62)	18 (41)	0.08
Right coronary artery	35 (50)	9 (35)	26 (59)	0.08

ECMO: extracorporeal membrane oxygenation; CPR: cardiopulmonary resuscitation; SD: standard deviation

### Direct transfer group

**Fig. 2:**
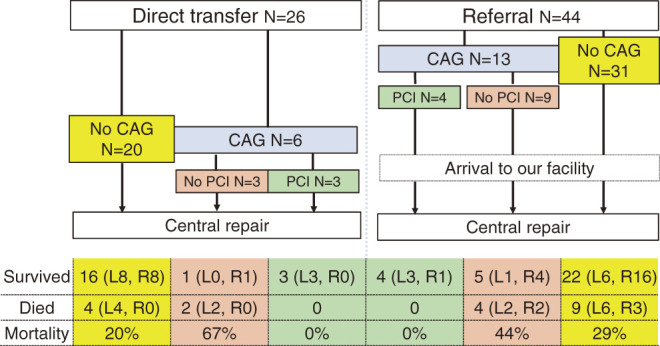
Patient flow diagram showing the relationship between catheter intervention and referral-related death. L: left coronary arterial malperfusion; min: minutes; R: right coronary arterial malperfusion

The direct transfer group included 26 patients (mean age: 66 ± 11 years) who were transported directly to our facility by ambulance. After arrival, the definitive diagnosis of AAAD was confirmed by routine echocardiography screening for ventricular wall motion, pericardial effusion, and intimal flap of the ascending aorta. If no evidence of AAAD was observed, emergent CAG was indicated for patients with AMI (n = 6, **Fig. 2**, blue column), which was usually followed by catheter interventions (n = 3, green column). In the remaining 3 patients, only diagnostic CAG was performed and subsequent PCI was not performed, mainly due to the unstable hemodynamics of the patients (orange column).

If AAAD was suspected during the screening, emergency computed tomography angiography was prioritized (n = 20, yellow column). All patients with suspected AAAD underwent emergency central aortic repairs.

**Fig. 3:**
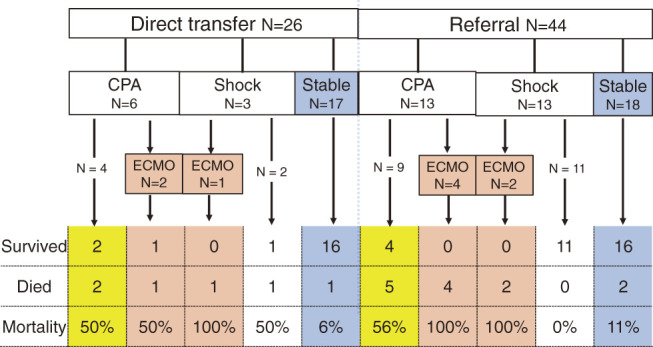
Flow chart of patients by hemodynamic status and mortality. ECMO: extracorporeal membrane oxygenation; CPA: cardiopulmonary arrest

**Figure 3** summarizes the surgical outcomes classified by preoperative hemodynamics. In the direct transfer group, 6 (35%) of the 26 patients had cardiopulmonary arrest (CPA) before or immediately after hospital arrival. Two of these patients were resuscitated with peripheral ECMO. In another patient in shock, peripheral ECMO was applied immediately after hospital arrival. The remaining 17 patients demonstrated stable hemodynamics (**Fig. 3**).

### Referral group

The referral group included 44 patients (aged 64 ± 15 years) with a definitive diagnosis of AAAD who were transferred from community hospitals. In all, 13 patients had undergone emergency CAG for suspected AMI without a definitive AAAD diagnosis (**Fig. 2**, blue column), and subsequent catheter intervention was performed in 4 patients (green column). In the remaining 9 patients, only diagnostic CAG was performed without subsequent PCI, mainly because of unsuccessful catheter insertion into the dissected coronary arteries (orange column). The other 31 (70%) patients with a definitive diagnosis of AAAD were transferred to our facility from referral hospitals (yellow column). All patients underwent emergency central aortic repairs.

As shown in **Fig. 3**, 13 (30%) of 44 patients required cardiopulmonary resuscitation (CPR) at the referral hospital, during transfer, or immediately after arrival. Peripheral ECMO was employed in 4 of the 13 patients during CPR at the referral hospital. Of the 44 patients, 13 (30%) were in a state of shock. Of these, peripheral ECMO was applied in 2 out of the 13 patients. The remaining 18 (18/44, 41%) patients had stable hemodynamics.

### All cohorts

The preoperative hemodynamic shock was noted in 35 (50%) patients, which occurs more frequently in the referral group than in the direct transfer group (59% vs. 35%, P = 0.049). The culprit vessel of coronary malperfusion was similar in the left and right coronary arteries between the 2 groups.

The in-hospital mortality rate in the entire study cohort was 27% (19/70). The in-hospital mortality rate was not significantly different between the 2 groups (direct transfer, 23%; referral, 30%, P = 0.59). The maximum postoperative levels of CKMB were 127 (31–448, IQR) and 156 (57–319, IQR) U/L in the direct transfer and referral groups. respectively (P = 0.74). Complications included postoperative respiratory failure, acute renal failure requiring dialysis, and new-onset permanent neurological deficits, which did not show significant differences between the 2 groups (**Table 2**).

**Table 2:** Postoperative outcomes

Variables	Overall (N = 70)	Direct transfer (N = 26)	Referral (N = 44)	P-value
Surgical results, n (%)				
In-hospital death	19 (27)	6 (23)	13 (30)	0.59
Incubation >72 hours	35 (50)	12 (46)	23 (52)	0.78
Tracheostomy	15 (21)	5 (19)	10 (23)	1
New-introduction HD	3 (4)	1 (4)	2 (5)	1
New-onset PND	6 (9)	1 (4)	5 (11)	0.65
Re-exploration for bleeding	21 (30)	9 (35)	12 (27)	0.41
Postoperative maximum of CK-MB (U/L), median (IQR)	156 (42, 357)	127 (31, 448)	156 (57, 319)	0.74
Mechanical circulatory support in total, n (%)	19 (27)	7 (27)	12 (27)	1
With IABP	10 (14)	4 (15)	6 (14)	1
With ECMO	14 (20)	5 (19)	9 (20)	1
With LVAS	3 (4)	2 (8)	1 (2)	1
With RVAS	2 (3)	0	2 (5)	1
Pacemaker implantation	15 (21)	5 (19)	10 (23)	1
ICU stay (day), median (IQR)	8 (4, 17)	9 (5, 19)	8 (4, 15)	0.66
Hospital stay (day), median (IQR)	32 (22, 47)	39 (28, 70)	30 (20, 46)	0.12

HD: hemodialysis; PND: permanent neurological deficit; IABP: intra-aortic balloon pumping; ECMO: extracorporeal membrane oxygenation; VAS: ventricular assist system; ICU: intensive care unit; IQR: interquartile range

In the direct transfer group, 19% (5/26) and 8% (2/26) of the patients required postoperative peripheral ECMO and ventricular assist device (VAD) support, respectively. In the referral group, 20% (9/44) and 7% (3/44) of the patients required postoperative peripheral ECMO and VAD support, respectively. The proportion of patients with low cardiac output syndrome that required postoperative mechanical circulatory support was not significantly different between the 2 groups (**Table 2**).

**Table 3** presents the shortlisted factors for in-hospital mortality. In the univariate analysis, the left coronary artery being the culprit vessel (P = 0.02, odds ratio [OR]: 4.0) and preoperative devastated status, such as the shock state, use of peripheral ECMO, and CPR. were identified as significant risk factors for in-hospital mortality. Moreover, diagnostic-only CAG without subsequent PCI tended to be identified as a risk factor (P = 0.06, OR: 3.5) (**Table 3**).

**Table 3:** Risk factors of In-hospital death

Variables	All patients (N = 70)	
	Univariate	
	P-value	OR (95% CI)
Age, ≥65	0.27	0.55 (0.2–1.6)
Male gender	0.87	0.91 (0.3–2.6)
Post history		
Hypertension	0.89	1.1 (0.2–6.2)
Hyperlipidemia	0.78	1.2 (0.4–3.5)
Ischemic heart disease	0.38	0.48 (0.1–2.4)
Old cerebral vascular accident	0.92	0.89 (0.1–9.1)
Preoperative status		
Shock	0.002	9.0 (2.3–34.9)
Under ECMO	0.001	36.4 (4.1–321)
Coma	<0.001	8.5 (2.5–28.4)
CPR	<0.001	10.8 (3.2–36.7)
Preoperative procedure		
CAG	0.61	1.4 (0.4–4.3)
Diagnostic-only CAG without subsequent PCI	0.06	3.5 (0.95–12.6)
Neck vessels dissection	0.47	0.55 (0.11–2.8)
Visceral malperfusion	0.06	9.4 (0.91–96.6)
The culprit of the left coronary artery	0.02	4.0 (1.3–12.8)
Via referral hospital	0.56	1.4 (0.5–4.3)

ECMO: extracorporeal membrane oxygenation; CPR: cardiopulmonary resuscitation; CAG: coronary angiography; PCI: percutaneous coronary intervention; OR: odds ratio; CI: confidence interval

### Preoperative CAG versus no CAG (**Fig. 2**, blue against yellow columns)

In both groups, 7 patients underwent CAG followed by a successful PCI (green column). Although all patients who underwent preoperative PCI survived, the diagnostic-only CAG without subsequent PCI (orange column) showed the highest mortality rate compared with immediate central repair (yellow column). One patient who underwent diagnostic-only CAG simultaneously with peripheral ECMO died in the hospital.

### Patient outcomes classified by preoperative hemodynamics (**Fig. 3**)

The in-hospital survival rate in patients with stable preoperative hemodynamics was 91% (32/35, **Fig. 3**, blue column). Among the patients with preoperative CPA but without preoperative peripheral ECMO support (yellow column), 46% (6/13) patients survived; only 1 (11%) of the 9 patients requiring preoperative peripheral ECMO support (red column) survived.

## Discussion

In Japan, malperfusion of the left coronary artery developed in approximately 3% of patients with AAAD.^7)^ Owing to its low incidence and significant in-hospital mortality risk, a consistent surgical approach for cases of malperfusion caused by AAAD still remains to be established.

The early reperfusion strategy has gradually received attention, and previous studies^1,8)^ have reported excellent surgical outcomes with this approach, implying that AMI treatment is essential for patients’ survival. However, in practice, early reperfusion with PCI completion for AMI as a bridge to central aortic repair can be interfered with by various medical, social, and/or graphical factors, and such a procedure is technically challenging.^9)^ In this study, the proportion of patients who underwent CAG followed by successful PCI was only 10% (7/70), indicating that only a few patients could benefit from early reperfusion in real practice. According to the heterogeneity of the clinical course from symptom onset to coronary reperfusion, the surgical data were analyzed, focusing on the appropriate timing of diagnosis, type of interhospital transportation, and reperfusion-related procedures.

In a community hospital without cardiovascular surgical services, difficulties are expected during the decision-making process. Based on the Japanese Registry of Acute Aortic Dissection,^10)^ nearly half of the Japanese patients with AAAD were transferred from community hospitals to comprehensive aortic centers after a definitive diagnosis. Thus, when cardiologists and/or emergency physicians encounter cases of myocardial ischemia caused by AAAD, deciding whether to proceed with initial treatment, including CAG, would be extremely challenging. In this study, 6 of the 12 patients who underwent preoperative CAG with unsuccessful reperfusion died in the hospital. All 6 required preoperative peripheral ECMO and could not be weaned off postoperatively, highlighting their severely compromised preoperative status. In addition, the median time of the interval from symptom onset to CPB establishment, which does not necessarily reflect the actual timing of reperfusion, was significantly longer in these patients than in the others (197 vs. 115 min, P = 0.03), indicating that delayed intervention is a potential factor contributing to poor outcomes. The expert opinion of the American Association for Thoracic Surgery^11)^ recommended a timely interhospital transfer for managing myocardial ischemia. The centralization of patients suspected of AAAD is critical, not only for central aortic repair but also for the initial diagnosis.

The reduced rate of preoperative shock status in the direct transfer group and the relevance between the hemodynamic status and in-hospital mortality led us to speculate that hemodynamic stability before central aortic repair should be prioritized. The application of preoperative mechanical circulatory support, including peripheral ECMO, was effective for patients with AMI-induced cardiogenic shock.^12)^ However, it has not demonstrated a survival advantage for AAAD. Yamasaki et al.^13)^ reported on 254 patients with out-of-hospital cardiac arrest caused by AAAD, in which ECMO was used in 26 cases; however, only 1 patient survived the procedure (1/26, 3.8%). In the present study, only 1 (11%) of 9 patients requiring preoperative peripheral ECMO support survived. Among these, all 6 patients in the transfer group experienced mortality. These results could indicate that peripheral ECMO, which relied on retrograde blood flow, may not be a definitive solution for improving clinical outcomes. Particularly in cases of severe coronary malperfusion due to aortic dissection, peripheral ECMO may not adequately restore coronary perfusion. Although peripheral ECMO for short-term hemodynamic stabilization to allow transfer to the operating room might be considered, these results could serve as a cautionary note, suggesting that prioritizing patient transportation may be more important than ECMO support, particularly if establishing peripheral ECMO took a significant amount of time.

A recent study reported the utility of active mechanical circulatory support with a percutaneous micro-axial flow pump, based on prioritizing left ventricular (LV) unloading for patients with AMI.^14)^ The ideal approach for AMI may target LV unloading and early reperfusion. Secure LV unloading needs to be an emergency operation as soon as possible given the nature of aortic dissection; however, in clinical settings, it would be very difficult because of several factors. These realities shaped our study design which sought to report on the actual management and outcomes in Japanese patients.

### Study limitations

Given the small number of participants in the present retrospective study, patient matching was not performed, and randomization was feasible in this single-center study. Data on the time course from the onset of aortic dissection during CAG at the referral hospital were varied in a few cases. Similarly, data on whether antithrombotic therapy was performed before CAG, including antiplatelet and anticoagulation treatments, were lacking. Moreover, owing to the difficulty of performing PCI in AAAD cases, the decision to perform PCI depended on each facility and cardiologist; therefore, selection bias was not entirely eliminated. To corroborate the results of this study, further studies involving numerous cases with patient-matched cohorts are needed.

## Conclusion

In patients with coronary malperfusion caused by AAAD, no significant difference was found in the mortality and morbidity rates according to the type of patient transportation. Regardless of the types of patients’ transportation, the use of preoperative peripheral ECMO itself could not be a definitive solution. Moreover, patients who eventually underwent CAG without subsequent PCI before central aortic repair may face fatal outcomes, especially when the culprit of the left coronary artery is suspected.

## Acknowledgments

We would like to thank Steve V from Enago (www.enago.jp) for the English language review.

## Declarations

### Ethics approval and consent to participate

Given the study’s retrospective nature, obtaining verbal or written informed consent from the patients was not required. Informed consent was obtained in the form of opt-out on the website. Those who rejected were excluded.

Reporting was approved by the National Cerebral and Cardiovascular Center Institutional Review Board (reference number: M30-057).

### Consent for publication

Informed consent for publication was obtained in the form of opt-out on the website. Those who rejected were excluded.

### Funding

This research did not receive any specific grant from funding agencies in the public, commercial, or not-for-profit sectors.

### Data availability

According to applicable data protection laws, the data that served as the basis for this article cannot be disclosed to the general public. Nonetheless, with the ethics committee’s approval, the data will be made available to the relevant author upon reasonable request.

### Author contributions

KN: Conceptualization; Data curation; Investigation; Methodology; Project administration; Visualization; Writing—original draft; Writing—review & editing.

YI: Conceptualization; Data curation; Supervision; Writing—review & editing.

YS: Writing—review & editing.HM: Conceptualization; Methodology; Supervision; Writing—review & editing.

All authors have read and approved the final version of the manuscript.

### Disclosure statement

The authors declare that they have no competing interests.

## Supplementary Material

Supplementary material 1 All variables examined in the logistic regression analysis.
