# Supplementary material for: Surgical Outcomes Stratified by Type of Transportation and Presence of Coronary Reperfusion in Patients with Coronary Malperfusion Caused by Type A Aortic Dissection
Source: Ann Thorac Cardiovasc Surg. 2025 Feb 1;31(1):24-00182. doi: 10.5761/atcs.oa.24-00182 (PMC11873597; doi:10.5761/atcs.oa.24-00182)
Supplement: Supplementary material 1 [file atcs-31-1-24-00182-s01.pdf]

Supplementary material 1. All variables examined in the logistic regression analysis.

Variables
Age
Gender
Body mass index
Post history
Hypertension
Hyperlipidemia
Ischemic heart disease
Old cerebral vascular accident
Diabetes mellitus
Chronic obstructive pulmonary disease
Chronic kidney disease
Preoperative status
Shock
Coma
Under peripheral extracorporeal membrane oxygenation
Cardiopulmonary resuscitation
Preoperative procedure
coronary angiography (CAG)
percutaneous coronary intervention (PCI)
Diagnostic only CAG without subsequent PCI
The culprit of left coronary artery
Via referral hospital
Intraoperative findings
Extracorporeal circulation time
Aortic cross-clamp time
Hypothermic circulatory arrest time
